# Supplementary material for: Changes in DNA Methylation in Response to 6-Benzylaminopurine Affect Allele-Specific Gene Expression in Populus Tomentosa
Source: Int J Mol Sci. 2020 Mar 19;21(6):2117. doi: 10.3390/ijms21062117 (PMC7139286; doi:10.3390/ijms21062117)
Supplement: Supplementary file 1 [file ijms-21-02117-s001.zip › supplementary -online/Supplementary Figure S1-S6.doc]

**Figure S1. Changes of WUE under long-term 6-BA treatment in *P. tomentosa*.** Wue is calculated by the following formula: WUE = Pn/ Tr.

**Figure S2. Statistics of transposons located in different genomic elements.** The circle on the left represents the classification of the siRNA in the intergenic region, and the circle on the right represents the classification of siRNA in the genic region. Red represents siRNAs related to transposons, the blue represents non-transposons.

**Figure S3. DNA methylation levels in different genomic features under control and 6-BA treatment.** The green line represents the total ratio of methylation sites in the control (CK) and 6-BA treatment groups. The red line represents the proportion of CHG methylation sites, and the blue line represents the proportion of CHH methylation sites.

**Figure S4. The expression levels of DNA methylation-related genes in control and 6-BA groups.** Error bars represent standard deviation (SD) of three biological replicates (n=3). Asterisks indicate significant differences between the 6-BA-treated and control groups (*P < 0.05, **P < 0.01). The red bars represent the 6-BA treatment group(6-BA), and the blue bars represent the control group(CK).

**Figure S5. Effects of different concentrations of 6-benzylaminopurine treatment on physiological and photosynthetic characteristics of *P. tomentosa*.** A–D Changes in total protein content, peroxidase (POD) activity, sucrose phosphate synthase (SPS) activity, and malondialdehyde (MDA) content in leaves at 0, 1, 10, 100, 1000μmol·L^-1^ of 6-benzylaminopurine treatment. And a, b, c represents grouping in multiple comparisons. The difference between groups of the same letter is not significant, while the difference between groups of different letters is significant (*P*<0.05).

**Figure S6. Pipeline for identifying allele-specific expression loci.** Raw reads were initially mapped onto the reference *Populus trichocarpa* genome. GATK software was used for transcriptome SNP calling.
